# Notes and News

**Published:** 1899-10-21

**Authors:** 


### Notes and News.

(Collected and Communicated.)

The Royal Orthopædic Hospital in Oxford Street has been undergoing alterations, and will shortly be re-opened.

The Lettsomian Lectures of the Medical Society of London will be delivered on March 5th and 19th, and April 2nd, 1900, by Sir William Mitchell Banks, LL.D., F.R.C.S., the subject being "Cancer of the Breast." The annual oration will be delivered on May 21st, 1900, by J. Kingston Fowler, M.D., F.R.C.P.

The vigilance of the medical officers for health, in respect to discovering food unfit for consumption, does not diminish. Thirteen hundredweight of horseflesh and diseased cows' flesh was recently seized by Dr. Warry at Hackney and destroyed. It was believed to be intended for sausage meat, but neither consigner nor consignee could be discovered.

The P. and O. Company's steamship "Peninsular," which arrived at Plymouth last Saturday from Bombay, had on board a patient suffering from bubonic plague, This was a Sidi boy who had been employed as a coal trimmer. He had reported himself sick on the 9th inst., the day after leaving Marseilles. The patient and his attendant were removed to the hospital ship "Pique," in Plymouth Sound, and the "Peninsular" proceeded on her way to London.

Railway speeds are always a matter of interest to medical men, partly because they have to travel much, partly because high speed has been thought by various observers to be a factor of importance in the production of some of the evil effects from which habitual railway travellers often suffer, but especially because high speeds, unless accompanied by very high-class carefulness, are apt to lead to serious accidents and to provide much surgical experience. It may, then, be worth recording that Mr. Robert Grimshaw, writing to the *Times*, states that the fastest mile of which he has any record was in May, 1893, on the New York Central, when the mile was run in 32 seconds, which equals 112½ miles an hour. He also says that five miles were made in three minutes, which equals 100 miles per hour. We know that some of our railways run fairly fast trains, but nothing like this. What would happen in a "smash" at 100 miles an hour seems as yet to be, fortunately, undecided.

In order to bring its meetings more in touch with the actual growing point of pathological investigation, the Pathological Society have determined that several of the meetings in each session shall take place at one or other of the various pathological laboratories which exist in London. There can be no doubt that this new departure will be a most valuable one.

It will be good news to many consumptive invalids to learn that a large sanatorium for consumptives will be open, probably in November, at Helouan, in Egypt. The building will be open to the desert on all sides, and the roof will be utilised as a winter garden. The window casements are to be constructed for the unrestricted admission of air. Separate rooms, with bath-rooms, are to be provided, and about 70 patients will be accommodated.

At last it looks as if shop assistants are to be allowed to secure a little rest whilst on duty. The Early Closing Association arranged a competition in model seats. Two hundred and twelve of these were shown at their premises, and the first prize was awarded for a seat which automatically disappears when released. All three prizes were awarded for seats with automatic action, this, of course, being essential where business must not be impeded. It is stated that thousands of orders have already been given for seats, and soon those establishments will have the first choice of efficient assistants where these most necessary conveniences are introduced.

The new children's ward at the York County Hospital has been opened by Mrs. Thynne. The foundation-stone was laid in 1897 by the Duke of Cambridge, the ward forming the Jubilee Memorial. It is called the Victoria. It provides accommodation for 18 children, and besides this there is a small isolation ward, a large day-room, and a ward kitchen, so that really a children's department is comprised under the less assuming name of children's ward. The building is on arches, and has a flat roof, so that it can be added to if desired. The walls are tiled to a height of five feet, above which is Parian cement. The total cost of the addition has been £6,025. The Jubilee collection will not suffice for this expenditure, but the Marriott Bequest Fund will probably be requisitioned as well.

The Society of Apothecaries has obtained the opinion of Sir E. Clarke, Q.C., M.P., on the Hunter case, and he is of opinion that the L.S.A. (1886) is entitled to describe himself as "Physician" or "Surgeon," or both, and that the Society of Apothecaries is justified in so advising its licentiates, notwithstanding the decision in the case of Hunter v. Clare. Sir E. Clarke is also of opinion that should the question be again raised in the Courts, the case of Hunter v. Clare would not of necessity be held to be a binding authority on this point, inasmuch as the conviction of the appellant was quashed on other grounds, and the opinions expressed by the Court as to the rights of the L.S.A. were, under the circumstances, immaterial to the issue. The Society of Apothecaries had previously obtained the opinion of Mr. Haldane, Q.C., M.P., that the decision of the Queen's Bench Division of the High Court was erroneous, and, in view of the opinions of these eminent counsel, the society has invited the co-operation of the General Medical Council in having the questions at issue retried, and the matter authoritatively set at rest.

